# Childhood Residential Mobility and Mental and Physical Health in Later Life: Findings From the Reasons for Geographic and Racial Differences in Stroke (REGARDS) Study

**DOI:** 10.1177/07334648231163053

**Published:** 2023-04-04

**Authors:** Irene H. Yen, Aleena Bennett, Shauntice Allen, Anusha Vable, D. Leann Long, Marquita Brooks, Robert K. Ream, Michael Crowe, Virginia J. Howard

**Affiliations:** 1 Public Health Department, 33244 University of California, Merced, CA, USA; 2 School of Public Health, Ryals Public Health Building 48653 University of Alabama, Birmingham, AL, USA; 3 Department of Family and Community Medicine, 8785 University of California, San Francisco, CA, USA; 4 School of Education, Sproul Hall, 8790 University of California, Riverside, CA, USA

**Keywords:** African Americans, housing, physical function

## Abstract

The study objective was to investigate the effects of childhood residential mobility on older adult physical and mental health. In REasons for Geographic and Racial Differences in Stroke (REGARDS) Study, we used linear regression models to investigate if number of moves during childhood predicted mental and physical health (SF-12 MCS, PCS), adjusting for demographic covariates, childhood socioeconomic status (SES), childhood social support, and adverse childhood experiences (ACEs). We investigated interaction by age, race, childhood SES, and ACEs. People who moved more during childhood had poorer MCS scores, *β* = −0.10, SE = 0.05, *p* = 0.03, and poorer PCS scores, *β* = −0.25, SE = 0.06, *p* < 0.0001. Effects of moves on PCS were worse for Black people compared to White people (*p* = 0.06), those with low childhood SES compared to high childhood SES (*p* = 0.02), and high ACEs compared to low ACEs (*p* = 0.01). As family instability accompanying residential mobility, family poverty, and adversity disproportionately affect health, Black people may be especially disadvantaged.

**What this paper adds**

• We found long-term health implications associated with frequent residential moves during childhood.
• African Americans, people whose families were low socioeconomic status or those experiencing early life adversity combined with frequent childhood residential moves, had synergistic negative effects on older adult health.
• Combinations of racialized identity, low socioeconomic status, or early life adversity with frequent childhood residential moves suggest social vulnerability with implications for older adult health.

**Applications of study findings**

• Policies that support housing stability, for example, eviction protections, for families are also policies that support older adults' health.
• Policies that support housing stability could be particularly impactful for older adult health among otherwise socially vulnerable groups.

According to the American Community Survey, approximately 40 million people in the US change residences each year, 13% of the population (Frost, 2020). The most common reason for moving is housing**-**related (40%) compared to 27% due to family and 21% due to jobs (Frost, 2020). Renters and low-income people are more likely to move than homeowners and high-income earners (Frost, 2020). Indeed, when and where low-income people move are often not of their choosing (DeLuca & Jang-Trettien, 2020; DeLuca et al., 2019). When and where low-income people move is in part due to a confluence of high housing costs and low supply of affordable units. High costs and low supply have created an urgent policy situation exacerbated by additional economic strain brought on by the COVID-19 pandemic. According to the Joint Center for Housing Studies, in 2004, one in three renter households paid more than 30% of their income on housing (Belsky et al., 2005) (spending over 30% of one’s income on housing is referred to as “rental cost burdened” or “rent burden”). Rent-burdened households are at a higher risk for eviction (Joint Center for Housing Studies, 2020).

Moving is disruptive, and if the impetus for the move is a negative, for example, job loss or eviction, moving could be interconnected with other precarity and instability. When households move, children might have to change schools (Been et al., 2011) and lose friends (Pettit, 2004){Ream, 2005}. Studying the effects of the instability is challenging because people moving frequently are harder to find and follow over time. And this would be compounded if people are poor. A body of social science research reports that high residential mobility combined with poverty has negative consequences during childhood which can have longer term effects, including poorer childhood adjustment (Adam, 2004), poorer self-regulation (Roy et al., 2014), and can catalyze other instabilities and lead to violence and victimization (Desmond et al., 2015).

Further, moving during childhood may affect children’s health and well-being and, in turn, could have downstream influences on health. Compared to participants who never had a residential move, a larger number of residential moves during childhood has been found to be associated with poorer childhood health (moved ≥3 times**)** (Busacker & Kasehagen, 2012), “deleterious” health outcomes in adulthood including greater risk of depressed affect, attempted suicide, smoking and alcoholism (moved ≥8 times**)** (Dong et al., 2005), and higher prevalence of many different psychiatric disorders (e.g., any personality disorders, broad and narrow schizophrenia, and any mood disorder (Mok et al., 2016). Much research in this area has focused on youth and young adulthood, and on psychopathology, substance abuse, and suicide (Webb et al., 2016).

We sought to add to the literature in this area to investigate if there are long-term health effects associated with childhood residential mobility and how racialized minority status, childhood socioeconomic status, and childhood adversity may affect the association between residential mobility and older adult health.

## Methods

We analyzed data from the REasons for Geographic and Racial Differences in Stroke (REGARDS) Study. More detailed descriptions of the study are available elsewhere (Howard et al., 2005). Briefly, REGARDS enrolled 30,239 community-dwelling individuals across the contiguous United States from January 2003 to October 2007. Participants were identified from commercially available lists and contacted via telephone. The study sampled adults at least 45 years of age and oversampled from the stroke belt (North Carolina, South Carolina, Georgia, Tennessee, Mississippi, Alabama, Louisiana, and Arkansas). The sample breakdown at baseline is 56% stroke belt residents, 42% Black people, and 55% women. Trained interviewers used computer-assisted telephone interviews (CATIs) to obtain verbal consent, measures of demographics, and relevant medical histories. At the initial in-person visit, participants were asked to complete a “Places You Have Lived Questionnaire” listing city, state, and age at the time of move for each location they lived for at least a year from birth until enrollment in REGARDS (Howard et al., 2010) (our IRB approval for this secondary analysis was UCM2018-198).

The two outcomes of interest were obtained during the enrollment CATI phone call: the Mental Health Component Score (MCS) and Physical Health Component Score (PCS). MCS and PCS were derived from the 12-Item Short Form Health Survey (SF-12). PCS and MCS are self-reported measures. The PCS, a quality of life measure, assesses whether physical health limits moderate activities (moving a table and climbing stairs), pain interfered with work, or activities were limited due to physical health. The MCS assesses if, due to emotional problems, the participant accomplished less than they would like or did work less carefully than usual; assess how much of the time the participant felt calm and peaceful, had a lot of energy, felt down-hearted, and if physical health or emotional problems interfered with social activities. Both measures have good reliability (two-week test–retest reliability ≥0.89) and validity (Ware et al., 1996). PCS and MCS are standardized to a mean score of 50 and standard deviation of 10, such that a score of 50 corresponds with the US average, and a 1-point difference is 1/10 of a standard deviation (Ware et al., 1996).

Using the group of participants who completed a valid “Places You Have Lived Questionnaire,” each unique location change (hereafter “move”) from age 6 to 18 was noted and counted. The number of residential moves is continuous and ranges from 0 to 12.

Demographic covariates include age at baseline, centered at 65, sex, race/ethnicity, income, and education. Baseline income was obtained on the CATI call and categorized less as than $20k, $20k–$34k, $35k–$74k, $75k and above, and Refused. Baseline education categories were derived from the baseline CATI call (Less than HS, HS, Some College, College+).

In July 2012, the ancillary study Childhood and Family Life Factors was initiated to collect childhood information through a mailed questionnaire to all active participants at the time (*n* = 20,671). This questionnaire included mother’s education, from which our measure of childhood socio-economic status (SES), was derived. High childhood SES was defined as a mother’s education level above 8^th^ grade (Vable et al., 2017). Adverse childhood events were summed into a continuous measure (range: 0–8). The eight adverse events were parental separation or divorce, parent remarriage, serious illness of a family member, death of a parent, witnessing domestic violence, substance abuse by a family member, loss of job by a parent, and having a parent go to jail (Dube et al., 2004). Childhood rurality at age 10 was based on the question “When you were 10 years old, did your family live in a rural area, a small town, large town, or a city?”. A measure of childhood social support was created based on a modified version of the ENRICHD Social Support Inventory (ESSI) survey {ENRICHD Investigators, 2001 #146}, which contained five questions with five point Likert scale responses ranging from “1-None of the time” to “5-All of the time.” The five questions were: “How often was there someone in whom you could talk to, trust and confide?”, “How often was there someone who showed you love and affection?”, “How often was there someone who could help you with your homework?”, “How often was there someone who encouraged and pushed you to succeed in school?”, and “How often did you have as much contact as you would like with someone you felt close to, someone in whom you could trust and confide?” Participants who score ≤2 on at least 2 of the 5 items and a total score ≤18 are considered to have low perceived childhood social support.

### Statistical Methods

**Figure 1.**
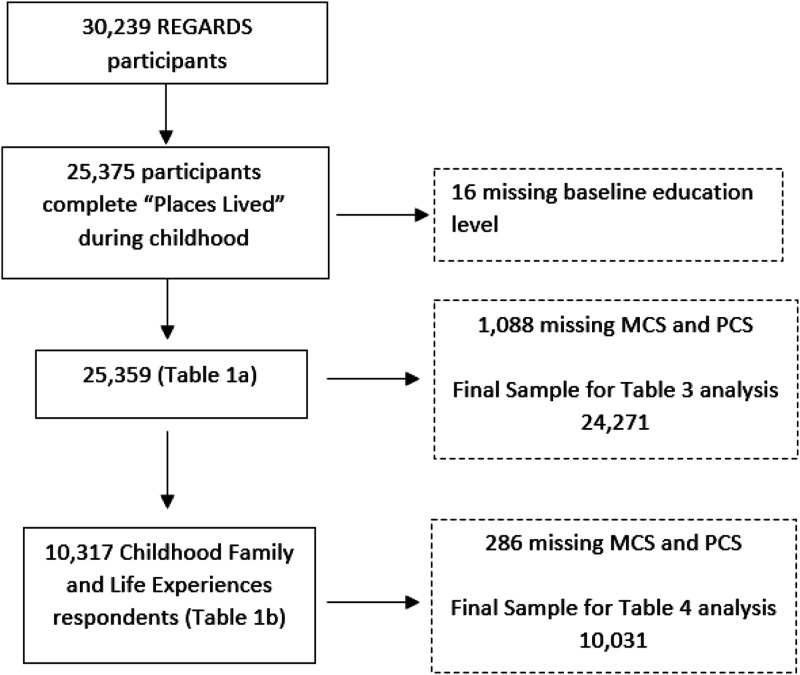
Flow Chart of Analytic Sample.

The association between childhood residential moves and MCS and PCS was modeled using linear regression. Model 1 included the exposure of interest, childhood moves. Model 2a adjusted for demographic factors. Model 2b included additional adjustment for SES variables. In the subset who responded to the Childhood and Family Life questionnaire only (*n* = 10,317), Model 2c additionally adjusted for childhood experiences. Interactions between the exposure and race, sex, and age, and in the childhood cohort only, childhood SES and ACEs were explored in Models 3, 4, 5, and 6, respectively. Specifically, we used statistical interactions to separately assess whether the associations between number of childhood moves and outcomes of MCS and PCS in the full model differed between Black versus White people, high childhood SES versus low childhood SES, and high number of ACEs (2 or more) compared to low number of ACEs (0 or 1). See Figure 1 for flow chart to show analytic samples.

There are 25,375 REGARDS participants with residential data for childhood, after removing 16 missing education, we were left with a sample of 25,359. For a sensitivity analysis, we investigated Models 1 and 2a–2c with the subset of participants who answered the childhood experiences questionnaire, for which Models 3–6 were conducted. The pattern of association was similar in both analytic samples.

Analyses were conducted using SAS, version 9.4 (SAS Institute).

## Results

Tables 1 and 2 show participant characteristics. The mean age of the respondents at baseline was 64.8, 44.5% female, and 38.1% Black people. The proportion of participant with 1 + moves who are Black is 32.2%, while 41.8% of the respondents with no moves are Black (Tables 1).

**Table 1.** Baseline Characteristics by Number of Childhood (Ages 6–18) Moves (N = 25,359).

	Overall	No Moves (*n* = 15,678)	1+ years With a Move (*n* = 9681)
Moves^1^	0.6 (1.1)	0 (0)	1.7 (1.1)
Age (**years)**^1^	64.8 (9.3)	64.5 (9.4)	65.3 (9.2)
Female	55.5	56.0	54.6
Black	38.1	41.8	32.2
Income			
<$20k	16.7	16.8	16.5
$20k–$34k	24.0	24.2	23.6
$35k–$74k	30.6	30.6	30.6
$75k+	16.6	16.2	17.3
Refused	12.1	12.1	12.0
Education			
Less than HS	10.8	10.3	11.6
High school	25.5	26.5	23.8
Some college	27.3	27.4	27.1
College +	36.4	35.7	37.5
SF-12^2^			
PCS	46.7 (10.5)	46.8 (10.3)	48.3 (9.8)
MCS	54.3 (8.3)	54.3 (8.3)	54.2 (8.2)

^1^Mean (SD) number of moves ranges from 0 to 12 (62% of participants had no childhood moves). Modeled continuously in regression.

^2^Mean (SD) ranges from 0 to 100.

**Table 2.** Baseline Characteristics by Number of Childhood (ages 6–18) Moves: REGARDS Childhood Family and Life Experiences Participants (N = 10,317).

	Overall	No Moves (*n* = 6356)	1+ years With a Move (*n* = 3961)
Moves^1^	0.7 (1.1)	0.0 (0.0)	1.7 (1.2)
Age (**years**)^1^	63.5 (8.5)	63.3 (8.5)	63.8 (8.5)
Female	55.9	56.0	55.9
Black	27.3	30.5	22.1
Income			
<$20k	10.2	10.3	10.1
$20k–$34k	21.6	21.7	21.5
$35k–$74k	34.8	35.0	34.5
$75k+	23.1	22.3	24.3
Refused	10.3	10.7	9.6
Education			
Less than HS	5.0	4.6	5.7
High school	22.2	22.9	21.0
Some college	26.5	27.2	25.5
College +	46.3	45.3	47.8
Low childhood SES	33.6	34.5	32.2
Urbanicity at 10 years age			
City	29.9	33.6	23.9
Large town	9.4	7.7	12.1
Rural area	30.5	30.5	30.5
Small town	29.8	27.8	33.1
Unknown	0.4	0.4	0.4
Childhood health			
Excellent	39.9	39.8	40.1
Very good	34.8	35.2	34.1
Good	18.9	18.7	19.2
Fair	5.3	5.1	5.6
Poor	1.1	1.1	1.1
Low social support	16.4	14.9	18.9
Number of adverse childhood events^1^	1.2 (1.4)	1.1 (1.3)	1.3 (1.5)
SF-12^2^			
PCS	48.6 (9.5)	48.8 (9.2)	48.3 (9.8)
MCS	55.0 (7.4)	55.0 (7.5)	54.9 (7.3)

^1^Mean (SD) number of moves ranges from 0 to 12 (62% of participants had no childhood moves). Modeled continuously in regression.

^2^Mean (SD) ranges from 0 to 100.

There are 10,317 participants with complete childhood data for childhood experiences and with historical places lived data (Table 2).

The demographic characteristics of the REGARDS subsample who filled out Childhood Family and Life questionnaire participant in 2012 include that their average age was 63.9, 30.4% of the participant were Black people, and 56.3% were female. A higher proportion of higher income and higher education people responded compared to the **overall** active REGARDS sample.

People who moved more during childhood had poorer MCS scores in later life, taking into consideration age, sex, race, income, and education, *β* = −0.10, SE = 0.05, *p* = 0.03 (see Table 3**).** We did not see this association differ by race/ethnicity or sex (race interaction *p*-value = 0.80, sex interaction *p*-value = 0.25). People who moved more during childhood had poorer PCS scores, and this association differed by race/ethnicity (race interaction *p*-value 0.06) and did not differ by sex (sex interaction *p*-value 0.30).

**Table 3.** Effect of Number of Childhood Moves on Older Adult Physical and Mental Functioning Among REGARDS Participants (N = 24,271).

	PCS			MCS		
	Beta	SE	*p*-Value	Beta	SE	*p*-Value
Model 1	−0.1209	0.0621	0.0515	−0.0316	0.0490	0.5185
Model 2a	−0.2433	0.0619	<.0001	−0.1060	0.0486	0.0292
Model 2b	−0.2513	0.0599	<.0001	−0.1023	0.0478	0.0325

Model 1: Number of childhood moves.

Model 2a: Model 1 + baseline age, sex, race.

Model 2b: Model 2a + income, education.

For the REGARDS participant subset who responded to the Childhood and Family Life Factors questionnaire, number of childhood residential moves did not predict poorer MCS in later life, taking into consideration age, sex, race, childhood SES, childhood social support, and ACEs, *β* = 0.03, SE = 0.06, *p* = 0.64. Number of years with a move did predict PCS in later life (see Table 4).

**Table 4.** Effect of Number of Childhood Moves on Adult Physical and Mental Functioning Among REGARDS Childhood and Family Life Factors Participants (N = 10,031).

	PCS			MCS		
	Beta	SE	*p*-Value	Beta	SE	*p*-Value
Model 1	−0.1249	0.0837	0.1358	−0.0679	0.0658	0.3022
Model 2a	−0.2096	0.0832	0.0118	−0.0760	0.0650	0.2423
Model 2b	−0.2280	0.0813	0.0051	−0.0654	0.0642	0.3082
Model 2c	−0.1839	0.0818	0.0247	0.0304	0.0646	0.6376

Model 1: Number of childhood moves.

Model 2a: Model 1 + baseline age, sex, race.

Model 2b: Model 2a + income, education.

Model 2c: Model 2b + childhood factors: cSES, self-reported urbanicity at age 10, self-reported childhood health, adverse events, childhood social support.

**Figure 2.**
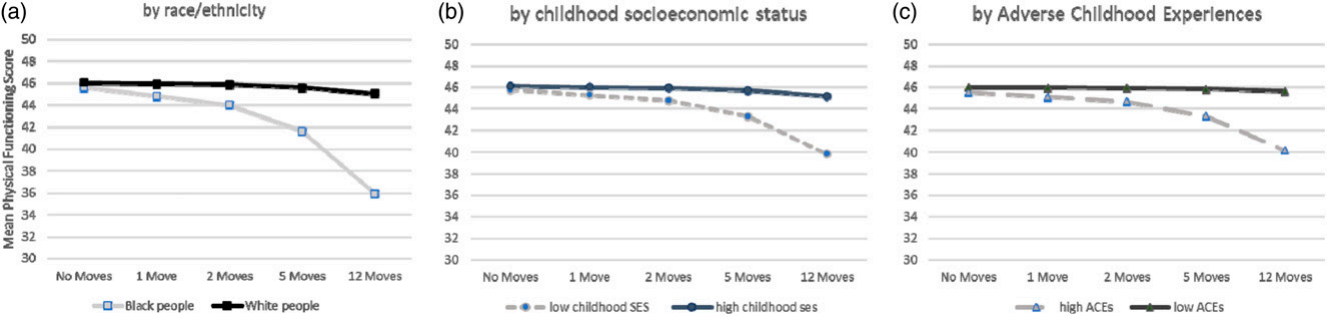
Childhood Moves Predicts Older Adult Physical-health Related Quality of Life (a) Model 3: Model 2b + race/ethnicity*moves. (b) Model 5: Model 2c + childhood SES*moves. (c) Model 6: Model 2c + ACEs*moves.

The effect of more moves during childhood on PCS differed by race (*p* = 0.002), childhood SES (*p* = 0.02), and ACEs (*p* = 0.01), but not sex (*p* = 0.54) (see Figure 2**)**. Black people with more childhood moves had poorer PCS compared to White people with the same number of childhood moves. Based on the model coefficients, a Black person who moved five times during childhood would have on average a 4.03 point lower PCS score as an older adult compared to a Black person who did not move.

Those who moved five times during childhood and whose family was low SES had on average a 2.47 lower PCS score as older adults compared to those who did not move and were also low SES. For people whose families were high SES, the average PCS score difference was 0.39, with non-movers having a higher PCS score as older adults.

People who experienced two or more ACEs and who moved five times during childhood had on average a 2.23 lower PCS score as older adults compared to people who did not move. For those who experienced 0 or 1 ACEs, people who moved five times had on average a 0.16 lower score than those who did not move during childhood.

## Discussion

Overall residential mobility has been on the decline in the US since the 1980s. However, who is moving and under what circumstances differs by race/ethnicity and socioeconomic status. Residential mobility is known to have health implications for children and adolescents (Busacker & Kasehagen, 2012) and is commonly associated with housing or work-related factors (Joint Center for Housing Studies, 2020). We sought to investigate if there are long-term health implications of residential mobility. REGARDs study participants were over age 45 at baseline in 2003–2007, children in the 1930s through the 1970s. We found different effects of childhood moves by race/ethnicity, childhood SES, and childhood adversities. Black people who moved more during childhood had lower physical functioning than whites with the same number of moves. Historically, Black people’s mobility has been constrained due to segregation and the factors that created segregated neighborhoods (South & Deane, 1993).

During the period when REGARDS participants were growing up, homeownership rates were rising from approximately 44% in 1940 to 63% in 1970 (US Census Bureau, 2000). Homeownership rates differ by race/ethnicity in the US, with the proportion of White people who are homeowners consistently being higher than the proportion of Black people {Haughwout et al., 2020 #170}. This period is also known for redlining practices such that Black families had restricted options for home purchasing as banks would lend to them only if they purchased in specific neighborhoods (Rothstein, 2018). Looking historically, from the mid-1980s to the mid-1990s, approximately 70% of White people were homeowners, while during the same period 42–44% of Black people were homeowners (Housing and Urban Development, 1997). The difference in home ownership—a principal asset that is central to the wealth portfolio of the average American {Oliver, 2013}—could be a factor in greater residential mobility during the REGARDs participants’ childhoods. Similarly, during this period, there was a difference in employment status between Blacks and Whites. From 1948 to 1972, Blacks experienced roughly twice the unemployment rate as Whites (Freeman, 1977).

We found that Black people who experienced more moves during childhood had poorer physical functioning when they were older adults compared to White people who experienced the same number of moves. Based on the model coefficients, a Black person who moved five times during childhood would have on average a 4.03 point lower PCS score as an older adult compared to a Black person who did not move. To put this finding in context, an analysis of the SF-36 measure (which is highly correlated with the SF-12 used here (Riddle et al., 2001)) found that a one point increase in the PCS was associated with 9% decrease in rate of hospital inpatient visits and 5% decrease in hospital outpatient visits (Kazis et al., 1989). Similarly, those whose families were lower SES during childhood experienced more negative effects of childhood residential mobility compared to those whose families were higher SES.

Information about childhood SES and ACEs was available for a subset of the REGARDS respondents, as 13, 210 people completed the Child Family and Life Questionnaire. When we run the same models described in left column of Table 2 for the Child Family and Life Questionnaire participant we see similar coefficients, though the MCS effects become statistically nonsignificant. Looking at who completed the question (table not shown) we know that a lower proportion of Black participant, a higher proportion of higher income respondents, and a higher proportion of participant with higher educational attainment responded. This suggests that people who moved more during childhood may be under represented in this subset, leading to a potential underestimate of the effects of childhood residential mobility.

There are a number of limitations to our analysis. The REGARDS study was designed to collect information from Whites and Blacks only. Other racial/ethnic groups were not included. Our data had a question ascertaining the number of location changes. We do not know the reasons for each move, and these could be different by sex, race, and other demographic factors. However, the large sample size, the national scope of the sample, and the high proportion of Black participant (by design) are all important strengths of this analysis to highlight.

We found that high residential mobility during childhood is associated with poorer mental and physical functioning in older adulthood. For Black people, people whose families were low SES during childhood, and those who experienced two or more ACEs, all showed poorer physical functioning compared to White people, people whose families were high SES during childhood, and those who experienced 0 or 1 ACEs, respectively. These interaction effects suggest that these combinations of characteristics are groups of people who are particularly socially vulnerable, that frequent mobility during childhood can have health impacts much later in life. As family instability accompanying residential mobility, family poverty, and adversity disproportionately affect health, Black people may be especially disadvantaged.
